# Treatment Planning Considerations for Robotic Guided Cardiac Radiosurgery for Atrial Fibrillation

**DOI:** 10.7759/cureus.705

**Published:** 2016-07-20

**Authors:** Oliver Blanck, Svenja Ipsen, Mark K Chan, Ralf Bauer, Matthias Kerl, Peter Hunold, Volkmar Jacobi, Ralf Bruder, Achim Schweikard, Dirk Rades, Thomas J Vogl, Peter Kleine, Frank Bode, Jürgen Dunst

**Affiliations:** 1 Department for Radiation Oncology, University Medical Center Schleswig-Holstein, Campus Kiel, Germany; 2 Saphir Radiosurgery Center, Frankfurt and Güstrow, Germany; 3 Robotics and Cognitive Systems, University of Lübeck; 4 Department for Radiation Oncology, Tuen Mun Hospital, Hong Kong, Hong Kong; 5 Institute for Diagnostics and Interventional Radiology, University Clinic Frankfurt, Germany; 6 Department for Radiology and Nuclear Medicine, Kantonsspital St. Gallen, Switzerland; 7 Radiology, Darmstadt, Germany; 8 Clinic for Radiology and Nuclear Medicine, University Medical Center Schleswig-Holstein, Campus Lübeck, Germany; 9 Institute for Robotics and Cognitive Systems, University of Lubeck,; 10 Institute for Robotics and Cognitive Systems, University of Luebeck, Institute for Robotics and Cognitive Systems, University of Lubeck; 11 Department for Radiation Oncology, University Medical Center Schleswig-Holstein, Campus Lübeck, Germany; 12 Department for Thoracic, Cardiac and Thoracic Vascular Surgery, University Clinic Frankfurt, Germany; 13 Cardiology Department, Sana Clinic Oldenburg in Holstein; 14 Department for Radiation Oncology, University Medical Center Copenhagen, Denmark

**Keywords:** atrial fibrillation, pulmonary vein isolation, cyberknife cardiac radiosurgery, stereotactic body radiation therapy, treatment planning, dose rate optimization, 4d dose calculation

## Abstract

**Purpose:**

Robotic guided stereotactic radiosurgery has recently been investigated for the treatment of atrial fibrillation (AF). Before moving into human treatments, multiple implications for treatment planning given a potential target tracking approach have to be considered.

**Materials & Methods:**

Theoretical AF radiosurgery treatment plans for twenty-four patients were generated for baseline comparison. Eighteen patients were investigated under ideal tracking conditions, twelve patients under regional dose rate (RDR = applied dose over a certain time window) optimized conditions (beam delivery sequence sorting according to regional beam targeting), four patients under ultrasound tracking conditions (beam block of the ultrasound probe) and four patients with temporary single fiducial tracking conditions (differential surrogate-to-target respiratory and cardiac motion).

**Results:**

With currently known guidelines on dose limitations of critical structures, treatment planning for AF radiosurgery with 25 Gy under ideal tracking conditions with a 3 mm safety margin may only be feasible in less than 40% of the patients due to the unfavorable esophagus and bronchial tree location relative to the left atrial antrum (target area). Beam delivery sequence sorting showed a large increase in RDR coverage (% of voxels having a larger dose rate for a given time window) of 10.8-92.4% (median, 38.0%) for a 40-50 min time window, which may be significant for non-malignant targets. For ultrasound tracking, blocking beams through the ultrasound probe was found to have no visible impact on plan quality given previous optimal ultrasound window estimation for the planning CT. For fiducial tracking in the right atrial septum, the differential motion may reduce target coverage by up to -24.9% which could be reduced to a median of -0.8% (maximum, -12.0%) by using 4D dose optimization. The cardiac motion was also found to have an impact on the dose distribution, at the anterior left atrial wall; however, the results need to be verified.

**Conclusion:**

Robotic AF radiosurgery with 25 Gy may be feasible in a subgroup of patients under ideal tracking conditions. Ultrasound tracking was found to have the lowest impact on treatment planning and given its real-time imaging capability should be considered for AF robotic radiosurgery. Nevertheless, advanced treatment planning using RDR or 4D respiratory and cardiac dose optimization may be still advised despite using ideal tracking methods.

## Introduction

Stereotactic Radiosurgery (SRS) has found its way into routine practice for cancer treatment even for some cardiac tumors [1-3]. A potentially non-cancerous indication for SRS is the treatment of cardiac arrhythmias [4] which has recently been investigated in animals [5-8] and human patients [9-11]. Atrial fibrillation (AF) is the most common cardiac arrhythmia [12] caused by aberrant electrical impulses originating mostly from the pulmonary veins (PVs) entering the left atrium (LA) [13]. A well-established approach for the treatment of paroxysmal AF patients is the electrical isolation of the PVs through catheter ablation [12], but SRS may potentially be used as a non-invasive alternative, especially for older patients. Locating and tracking the heart with SRS, however, remains challenging [5-8]. The first system to be used in animals and humans [5, 6, 9-11] was the CyberKnife® (Accuray Inc, Sunnyvale, USA) [14], where gold markers (fiducials) were implanted close to the lesion and used for target tracking during treatment with high dosimetric accuracy despite respiratory and cardiac motion [15].

While some groups are investigating complete non-invasive cardiac SRS with carbon ions [16, 17] or MRI-Linear-Accelerators [18], our goal was to analyze the general feasibility of cardiac SRS for paroxysmal AF in humans using treatment planning simulations and include possible options without fiducial implantation for the CyberKnife. The optimal non-invasive tracking method would be completely marker-less [19] which is already available for lung tumors for the CyberKnife [20]. A second option is the integration of an ultrasound tracking system into the CyberKnife [21, 22], with the downside that radiation beams need to avoid the ultrasound probe on the patient’s chest. A third, minimally invasive option may be the use of a single catheter, which may temporarily be attached to the right atrial septum [10, 11]. Despite the advantage of having a pseudo fiducial near the PVs, a downside may be significant differential motion especially compared to the left PVs [23].

## Materials and methods

Multiple data collection studies were performed to generate the presented data in various locations. All data were approved by the local ethics committees and performed under patient consent. The Institutional Review Board (IRB) of the University of Frankfurt and IRB of the University of Lübeck approved the collection of data for this study.

### Anatomical considerations

**Figure 1:**
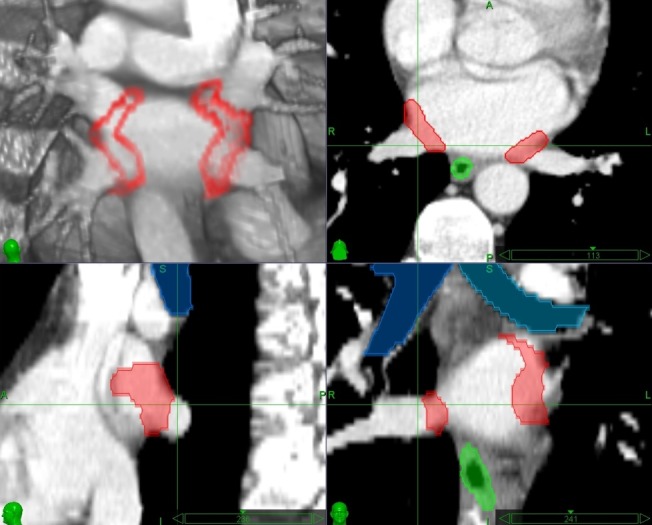
Contours for a theoretical radiosurgery treatment for atrial fibrillation Red = radiosurgery lesion, green = esophagus, blue = bronchial tree

The target contours for paroxysmal AF SRS will cover the left atrial-venous wall, the myocardium and the myocardial sleeves of the PVs transmural at the PV antrum, similar to catheter ablation [12, 13, 24-29]. The contours will be approximately 4-6 mm wide along the PVs / LA and approximately 2-4 mm deep depending on the tissue thickness (Figure 1). Typical PV anatomy with four distinct PV ostia is present in approximately 40% of the population [24-29]. Abnormalities of PV anatomy include the presence of a common left trunk or variations of right middle PVs.

Besides intra-cardiac structures, the organs at risk (OAR) closest to the target lesions are the esophagus, the bronchial tree, and the aorta. Especially the location of esophagus seems significantly variable along the PV-LA wall [30-34]. A pre-validation contouring study based on 133 patients (*unpublished*) has demonstrated that the location of the esophagus was posterior to the right inferior PV ostium in 10.5%, central and close to the right PV ostium in 9.0%, central and away from any PV ostium in 19.6%, central and close to the left PV ostium in 19.6%, and posterior to the left inferior PV ostium in 41.3% of the patients, agreeing well with the literature [30-34]. To summarize, in approximately 50% of the patients the esophagus is in direct contact with the actual target lesion, ruling out those patients for AF radiosurgery. Furthermore, some studies suggest that the esophagus may move along the PTV-LA wall [35], but conclusive results on the cause and frequency remain unpublished. A pre-validation set up study for cardiac SRS in 15 healthy volunteers on daily repeated breath-hold cardiac MRI (*unpublished*) demonstrated at least that the esophagus location at the left atrium differed by an average of only 1.5 mm (range, 0.7-2.6 mm). To date, significant location variability not caused by respiratory motion has not been reported in radiotherapy or radiosurgery literature.

### General treatment planning for cardiac radiosurgery

All lesions and OAR contours presented in this study were guided by surgeons and cardiologists experienced in AF treatment and radiation oncologists experienced in radiosurgery, importantly all being experienced in experimental cardiac radiosurgery. Initial lesion contouring was performed with the CardioPlan® Software (CyberHeart Inc, Sunnyvale, USA) and an isotropic margin of 3 mm was added to generate the Right and Left Planning Target Volumes (RPTV / LPTV) based on the accuracy of the CyberKnife [14], and previous studies [1-3, 5, 6, 9-11, 15]. An extra margin for the cardiac motion was not generated by the cardiac motion at the PV antrum is small [5-7, 18, 21, 23] and has a likely neglectable impact on the dosimetry [15, 36].

OAR contouring, as well as treatment planning, was performed with the MultiPlan® Software (Version 4.x, Accuray) with final Monte Carlo dose calculation [37] and density overwrites of the contrast enhancement in the heart on the planning computed tomography (CT). Sequential Multi-Objective Optimization (SMOO®) [38] was performed using the Iris® collimator [39] according to the best practice guidelines for robotic radiosurgery treatment planning [40]. Prescription dose was 25 Gy in a single fraction to the LPTV and RPTV according to results known prior to this study [5, 6]. OAR limits for this study were selected according to published literature at the time of this study (Table 1) [41, 42].

**Table 1:** Dose constraints for close organs at risk for atrial fibrillation radiosurgery Grading based on CTCAE (version 4.03) and single fraction dose limits based on [41, 42] DMax = Maximum Dose, VxGy = Volume receiving X Gy or more

Organ at risk	Side Effect Risk	Dmax	Volume
Esophgaus	<= Grade 1	< 14 Gy	V9.0Gy < 1 cc
	>= Grade 3	> 19 Gy	V14.5Gy > 5 cc
Brochial Tree	<= Grade 1	< 14 Gy	V10.0Gy < 1 cc
	>= Grade 3	> 22 Gy	V10.5Gy > 4 cc
Coronary Artery	<= Grade 1	< 16 Gy	No data
	>= Grade 3	20Gy Circumferential	
Major Vessels	<= Grade 1	No data	
	>= Grade 3	> 37 Gy	V31Gy > 10 cc

### Treatment planning under ideal conditions

An ideal condition would be to track each target (RPTV and LPTV) directly via the already existing CyberKnife marker-less tracking system for lung tumors (XSight® Lung, Accuray) [20]. To initially analyze the feasibility of human AF radiosurgery under such ideal conditions, we obtained 18 planning CTs under informed consent from patients undergoing AF ablation or lung cancer radiosurgery which had a favorable esophagus location (away from any PV ostia). Based on the planning CTs we then generated treatment plans with the above-mentioned method of contouring and optimization.

### Dose rate optimization under ideal conditions

Based on previously performed animal studies [5, 6] we found that for a given region the maximum total dose delivered over a certain time, further denoted as regional dose rate (RDR), is an important factor for AF radiosurgery, especially for the prolonged treatment times with the CyberKnife. While extended pulsed irradiation seems to have no negative effect on tumor cell destruction [43, 44], healthy cells which are targeted in AF radiosurgery may start their repair progress as soon as 20 min after first irradiation [45, 46].

**Figure 2:**
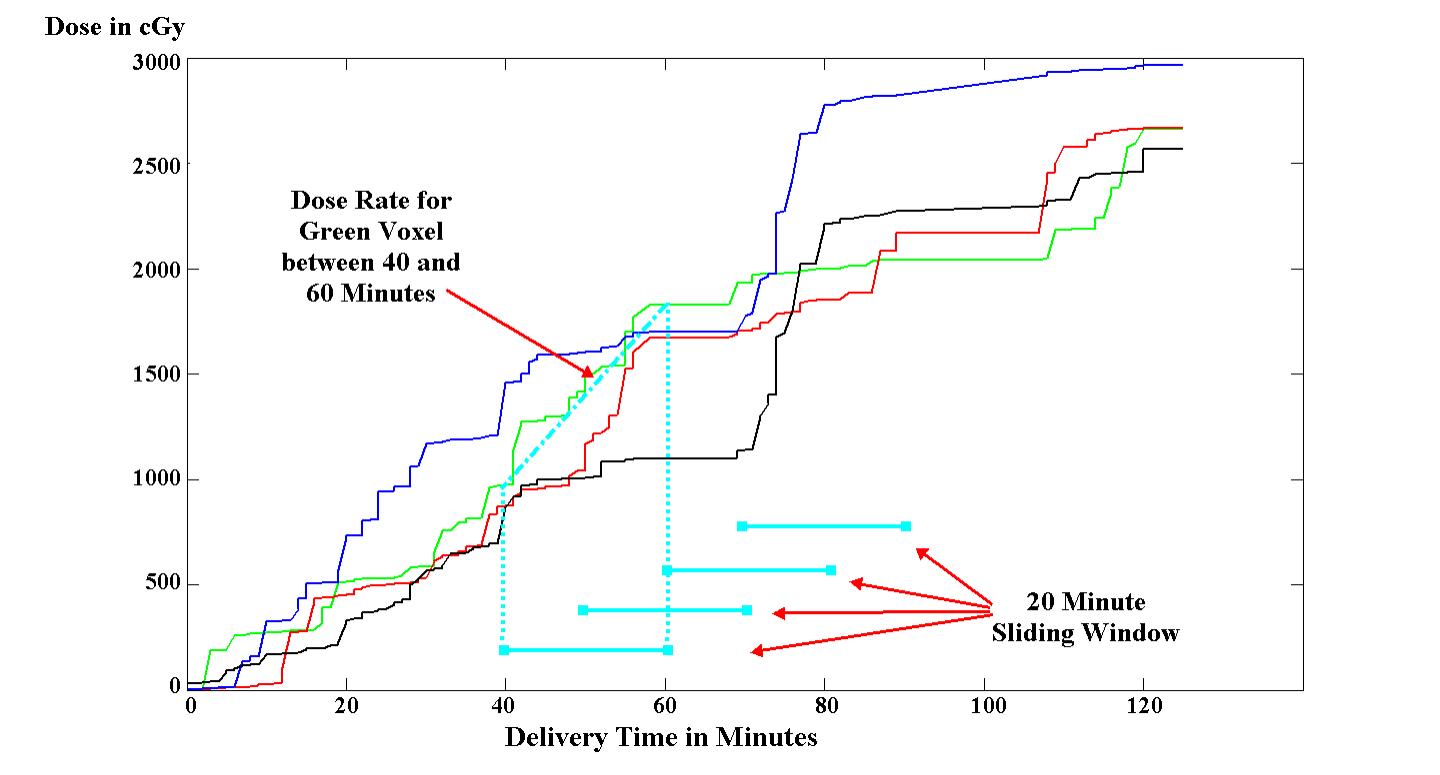
Principles of the CyberKnife regional dose rate calculation Calculation (cyan) for four different target voxels (blue, green, red, and black line) of an example atrial fibrillation radiosurgery treatment plan.

To analyze impacts on prolonged treatment times for CyberKnife AF radiosurgery we estimated the RDR for 12 of the 18 cases with standard CyberKnife beam delivery sequence over a given time window (*RDR_t_minutes_*) by a) calculating the cumulative dose for each PTV voxel at every minute during the projected treatment and b) finding the maximum dose difference between any two time points in the treatment course *a* and *a-t* minutes (Figure 2). The dose calculation was performed using an in-house planning system (experimental CyberKnife Planner, eCKP, version 2) and the projected treatment time was calculated using the formula: Estimated Fraction Treatment Time (EFTT, in minutes) = Robot and Iris Motion Time (number of nodes / 4 + number of beams / 20) + Beam On Time (MU / CyberKnife Dose Output) + Imaging Time (number of beams / number of images per beam / 10) + Synchrony Time (number of beam / 15). EFTT does not include patient setup.

**Figure 3:**
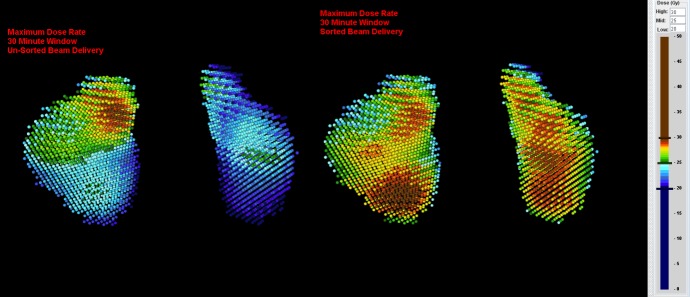
Regional dose rate calculation without (left) and with (right) beam delivery re-sorting Voxel display of left and right planning target volume (PTV) with our in-house planning system (eCKP). Note that in the unsorted beam delivery only 47.3% (RPTV) and 3.4% (LPTV) of the voxels and in the re-sorted beam delivery 87.4% (RPTV) and 89.0% (LPTV) of the voxels receive 25 Gy in less than 30 minutes (estimated fraction treatment time = 42 minutes).

In order to evaluate potential RDR increase of sequential beam-to-target delivery, we re-sorted the beam delivery sequence into four consecutive phases so that first all beams hitting only the RPTV were delivered, then all beams hitting the RPTV first and the LPTV second, followed by all beams hitting the LPTV first and the RPTV second, and finally all beams hitting only the LPTV. We then re-evaluated the RDR for the re-sorted beam delivery sequence and compared the results to the RDR of the original beam delivery sequence. Note that the overall treatment may take longer since the robot has to travel between re-sorted nodes. An example calculation is presented in Figure 3.

**Figure 4:**
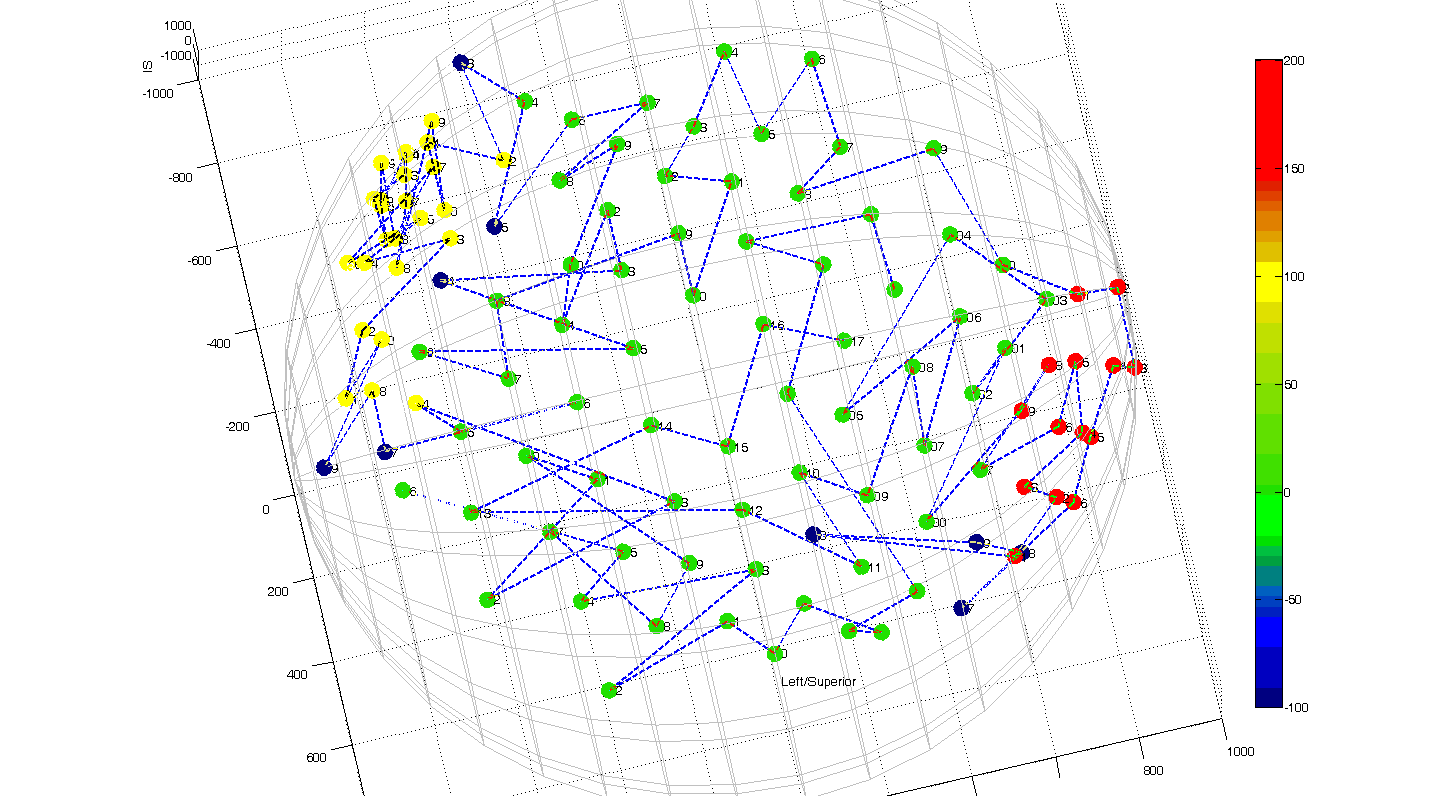
CyberKnife atrial fibrillation node set for optimized regional dose rate delivery Nodes presented as colored dots on a hemisphere: green dots = anterior node set, red dots = left lateral node set, yellow dots = right lateral node set, blue dots = removed nodes for optimized path traversal

To reduce the problem of low RDR with standard CyberKnife beam delivery during optimization, the following procedure was followed. We divided the original full node set into three separate nodes sets (anterior, left lateral and right lateral node set, Figure 4) and re-planned the original cases (without changing the SMOO optimization scripts) by targeting the RPTV from the anterior and the right lateral node set and the LPTV from the left lateral and the anterior node set (node set delivery sequentially in that order). We re-evaluated the RDR for those cases and compared the results to the other beam delivery sequences. Note that by splitting the node sets in lesion specific node sets, separate lesion tracking also becomes possible, thus reducing possible effects from differential motion of the RPTV and LPTV.

### Treatment planning under ultrasound guidance

Since direct tracking of the target lesions may be difficult solely based on x-ray imaging, ultrasound guidance may overcome the limitation of lesion visibility and also of non-continuity of location information from sporadically taken x-rays. Ultrasound tracking for the beating heart has already been investigated [21], and active ultrasound tracking (beam steering with an ultrasound signal) for the CyberKnife (or other radiotherapy systems) seems feasible [22, 47, 48]. One drawback of ultrasound tracking is the relatively large ultrasound probe on the patient’s chest. Radiation beams passing through the probe may significantly alter the calculated dose distribution and may also lead to hardware failures inside the probe. Consequently, those beam directions have to be blocked for CyberKnife AF radiosurgery.

To analyze the effect on plan quality difference when blocking the beam paths through an ultrasound probe, we estimated the ultrasound probe position for an optimal ultrasound window on the planning CT using in-house software (Institute for Robotics and Cognitive Systems, Lübeck, Germany) and inserted the resulting probe position as a contour into four patient treatment plans. We then compared the quality of the plans optimized with the same repeated procedure as described above with and without blocking the ultrasound probe.

### Treatment planning under temporary fiducial guidance

Since marker-less tracking on x-ray may be challenging, and ultrasound tracking is still under investigation, a compromise solution to treat AF with the CyberKnife may be the use of a “temporary fiducial” (i.e., a catheter) close to the lesion. As opposed to invasively implanting fiducials [6, 7, 9], this procedure is only minimally invasive and has been investigated in a single human treated for cardiac arrhythmia [10, 11], however not for AF. A potential location for an AF radiosurgery guiding catheter would be the atrial septum in the right atrium, mainly to avoid the crossing into the left atrium which may bear significant risks for the patient [12]. However, the right and left PVs in humans may move independently with respect to each other [23] and with respect to the atrial septum which necessitates some form of compensation for AF radiosurgery. For this purpose, we collected 4D cardiac gated contrast-enhanced CTs at regular breathing end expiration and end inspiration under informed consent of four patients undergoing cardiac CT, again with favorable esophagus location.

Initially, we generated a static plan based on the mid-diastole phase of the end expiration CT repeating the contouring and optimization procedures as described above. We then defined a likely possible catheter position at the atrial septum in the right atrium to be further used as reference tracking point for CyberKnife 4D dose calculation [49] for the expiration and inspiration CT in the same cardiac phase. Deformation modeling for 4D dose calculation was supported by multiple landmarks [50] and we compared static planning vs. 4D dose calculation of the static plan and 4D dose optimization, similar to our previous study for lung tumors [51]. Previous studies suggest that the impact of cardiac motion on the treatment planning is small with likely neglectable impact on the dosimetry [5-7, 15, 18, 21, 23, 36]. Since we had the necessary data for simulation, we also compared static planning vs. 4D cardiac dose calculation of the static plan based on the 4D cardiac CT in end expiration.

## Results

### General treatment planning for cardiac radiosurgery

**Figure 5:**
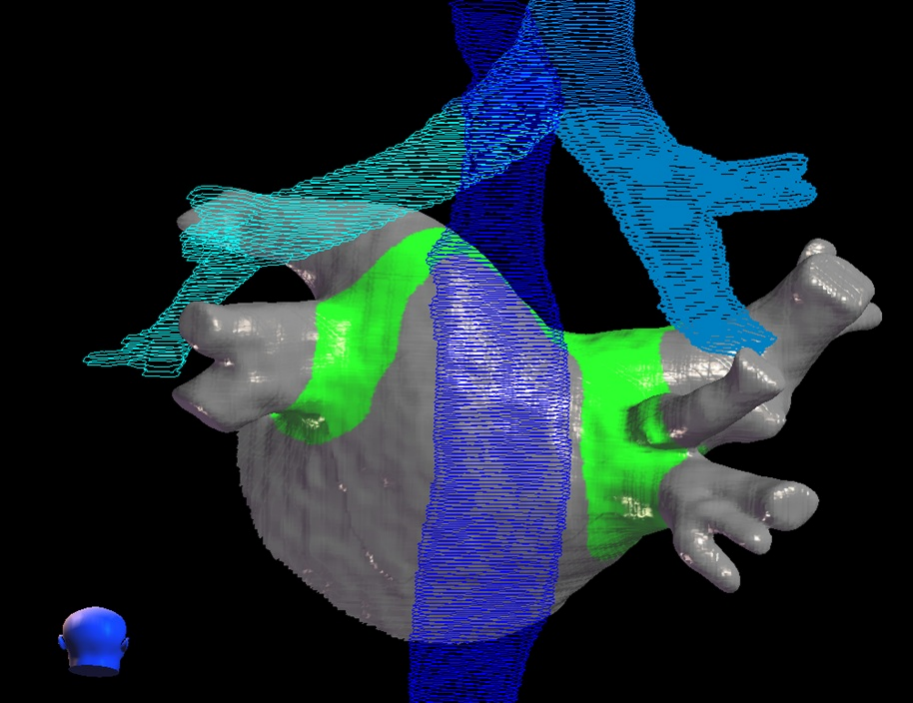
Example of an atrial fibrillation radiosurgery treatment plan in CardioPlan® (CyberHeart, USA) Dark blue = esophagus, cyan = bronchial tree, green = 25 Gy prescription dose mapped onto the left atrium

**Figure 6:**
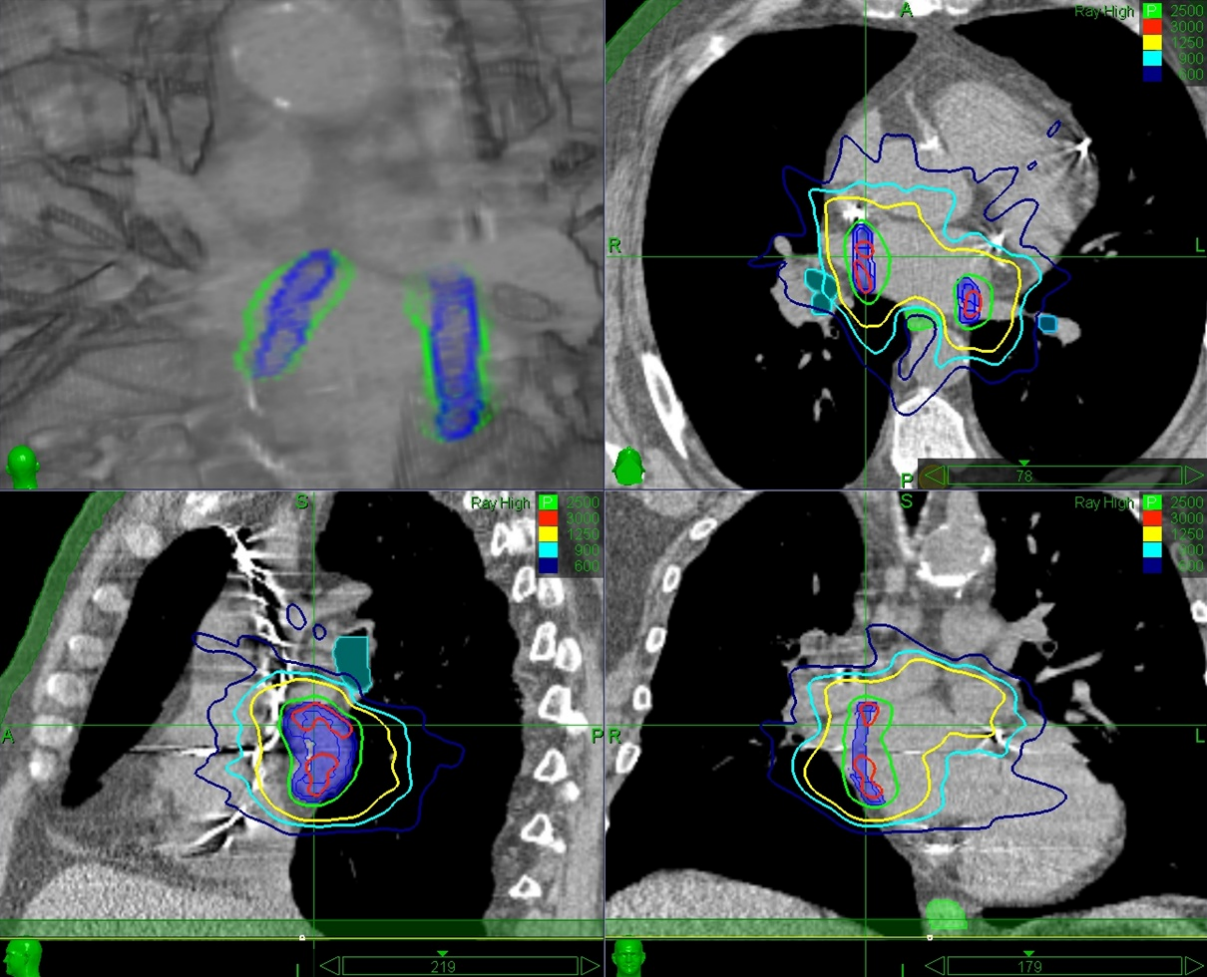
Example of an atrial fibrillation radiosurgery treatment plan in MultiPlan® (Accuray, USA) 3D dose overlay (top left), axial (top right), sagittal (bottom left) and coronal (bottom right) view Blue = planning target volume, green = esophagus, cyan = bronchial tree Isodose lines = 30 Gy (red), 25 Gy (green), 12.5 Gy (yellow), 9 Gy (cyan), 6 Gy (blue)

Median right PV lesion and left PV lesion volumes ranged from 4.6 cc to 18.0 cc (median, 9.8 cc) and from 4.2 cc to 11.9 cc (median, 6.9 cc), respectively, resulting in PTV volumes ranging from 17.0 cc to 49.1 cc (median, 31.0 cc). Since electrical isolation of the pulmonary veins has to be complete, we prescribed 25 Gy to ≥ 99% coverage of the PTV to a median isodose of 80% (Table 2). Maximum doses for the esophagus and the bronchial tree were high for a single fraction treatment (median, 16.4 Gy, and 17.6 Gy, respectively) and we failed to meet the dose constraints for grade three side effects in one case for the esophagus and in three cases for the bronchial tree (overall, in 22.2% of the cases). The low lung volume doses seem to be of no concern as opposed to the high heart volume doses (median V_10Gy_, 15.6% of the whole heart). Estimated fraction treatment time ranged between 50 and 87 min (median, 61 min). An example of an AF radiosurgery treatment plan is presented in Figures 5-6.

**Table 2:** Atrial fibrillation radiosurgery treatment planning results under ideal conditions LC = Lung Cancer, AF = Atrial Fibrillation, PTV = Planning Target Volume, LCA = Left Coronary Artery, MU = Monitor Units, EFTT = Estimated Fraction Treatment Time, DMax = Maximum Dose, VxGy = Volume receiving X Gy or more

			PTV	Esophgaus		Bronchial Tree		LCA	Lung	Heart				
		Rx	Coverage	DMax	V9	DMax	V9	DMax	V10	V10				EFTT
Patient	Case	(%)	(%)	(Gy)	(cc)	(Gy)	(cc)	(Gy)	(%)	(%)	Nodes	Beams	MU	(min)
1	LC	81%	99.2%	18.1	0.8	14.5	0.1	10.8	1.5%	16.5%	54	84	27733	56
2	LC	80%	99.5%	15.4	0.6	15.3	1.3	11.3	1.4%	15.0%	46	107	23964	58
3	AF	80%	99.4%	15.3	1.1	17.9	2.0	14.0	1.0%	14.6%	67	135	35479	87
4	LC	81%	99.1%	16.2	0.6	15.1	0.9	13.6	0.8%	14.7%	55	107	28904	60
5	LC	78%	99.2%	16.4	1.3	17.8	0.8	8.0	0.9%	17.0%	55	95	29490	64
6	AF	80%	99.2%	15.2	0.3	15.7	0.4	10.0	1.3%	18.8%	65	110	32885	65
7	LC	80%	99.4%	14.5	0.9	16.9	1.1	13.2	1.0%	11.8%	51	127	31564	72
8	AF	79%	99.4%	17.1	1.2	17.7	0.8	14.1	0.9%	14.6%	66	104	25443	62
9	AF	80%	99.2%	16.4	3.9	18.5	7.0	13.3	1.8%	17.8%	59	109	30582	60
10	AF	80%	99.5%	17.1	1.9	17.8	1.4	13.5	1.7%	14.9%	49	88	25318	53
11	AF	78%	99.0%	15.2	0.8	19.4	2.4	14.5	1.5%	18.0%	56	117	33727	67
12	AF	81%	99.2%	16.3	1.6	18.4	3.4	13.6	0.9%	16.2%	45	89	22579	50
13	AF	79%	99.1%	16.8	2.6	18.5	5.4	14.0	2.1%	18.3%	59	105	27107	59
14	LC	81%	99.2%	16.9	1.4	17.9	4.7	14.1	0.7%	12.8%	51	93	22967	56
15	LC	79%	99.1%	14.6	0.8	17.0	0.9	13.4	0.7%	9.6%	54	119	31440	73
16	LC	77%	99.1%	20.0	4.4	19.7	1.5	14.8	1.3%	17.3%	53	145	37066	82
17	LC	77%	99.2%	18.8	1.9	19.5	1.7	15.3	1.3%	21.3%	55	156	35920	82
18	LC	80%	99.5%	18.5	0.4	18.5	2.0	13.2	1.7%	13.5%	48	84	23211	55
Max		81%	99.5%	20.0	4.4	19.7	7.0	15.3	2.1%	21.3%	67	156	37066	87
Min		77%	99.0%	14.5	0.3	14.5	0.1	8.0	0.7%	9.6%	45	84	22579	50
Median		80%	99.2%	16.4	1.2	17.9	1.5	13.5	1.3%	15.6%	54.5	107.0	29197	61.0
Average		80%	99.2%	16.6	1.5	17.6	2.1	13.0	1.2%	15.7%	54.9	109.7	29188	64.5

### Dose rate optimization under ideal conditions

The initial calculation of the regional dose rate (RDR) over 40-50 min windows demonstrated that the RDR coverage was as low as 10.1% for the RPTV (median, 48.7%) and 3.2% for the LPTV (median, 61.5%). This translates into the fact that with regular CyberKnife beam delivery only approximately half of the PTV voxels receive the prescribed dose of 25 Gy in more than 45 min. Re-sorting the beam delivery sequence resulted in an increase of the median RDR coverage of 42.2% for the RPTV (90.9%) and 30.1% for the LPTV (81.6%). However, lowest RDR coverage was still found to be only 50.8% for the LPTV in one case. Using the divided node set during optimization, the median RDR coverage was found to be similar to the re-sorted beam delivery at 94.5% for the RPTV and 91.1% for the LPTV, but now RDR coverage was above 80% for all cases. This finding translates into the fact that with a divided node set, more than 80% of the PTV voxels receive the prescribed dose of 25 Gy in less than 45 min. This is a large improvement given known cell repair rates [45, 46]. The overall median treatment time was 4.5 min and 2.5 min longer than regular beam delivery for the re-sorted and the divided node set, respectively. Details are presented in Table 3.

**Table 3:** Regional dose rate optimization results RPTV = Right Planning Target Volume, LPTV = Left Planning Target Volume,  EFTT = Estimated Fraction Treatment Time RDR = Regional Dose Rate (RDR window was selected based on EFTT of the initial treatment plan)

		Regular Beam Delivery				Re-Sorted Beam Delivery			Divided Node Set Delivery			
	RDR			RDR Coverage			RDR Coverage				RDR Coverage	
	Window		EFTT	RPTV	LPTV	EFTT	RPTV	LPTV		EFTT	RPTV	LPTV
Patient	(min)	Beams	(min)	(%)	(%)	(min)	(%)	(%)	Beams	(min)	(%)	(%)
1	45	84	56	48.0%	64.1%	61	89.9%	97.7%	83	58	94.6%	97.2%
2	45	107	58	56.7%	41.0%	63	89.1%	92.3%	101	60	89.9%	89.3%
4	45	107	60	33.5%	66.8%	65	87.8%	92.9%	100	68	87.6%	93.1%
5	50	95	64	81.6%	68.5%	68	97.8%	75.5%	92	66	97.8%	84.6%
6	50	110	65	49.3%	84.1%	71	90.9%	94.9%	130	72	90.9%	90.1%
8	50	104	62	15.3%	58.8%	68	84.6%	89.6%	101	68	94.3%	93.3%
9	45	109	60	27.2%	3.2%	65	90.9%	91.8%	112	62	90.4%	95.6%
10	40	88	53	68.2%	55.4%	57	92.6%	50.8%	92	55	95.5%	83.5%
11	50	117	67	74.0%	64.3%	74	98.3%	89.2%	110	69	98.4%	90.5%
12	40	89	50	54.7%	29.7%	56	89.2%	75.6%	85	51	98.4%	91.7%
13	45	105	59	29.6%	36.0%	63	93.2%	91.5%	115	62	93.8%	88.6%
14	45	93	56	10.1%	76.7%	61	95.3%	92.7%	88	57	96.4%	94.9%
Max	50	117	67	81.6%	84.1%	74	98.3%	97.7%	130	72	98.4%	97.2%
Min	40	84	50	10.1%	3.2%	56	84.6%	50.8%	83	51	87.6%	83.5%
Median	45.0	104.5	59.5	48.7%	61.5%	64.0	90.9%	91.6%	100.5	62.0	94.5%	91.1%
Average	45.8	100.7	59.2	45.7%	54.1%	64.3	91.6%	86.2%	100.8	62.3	94.0%	91.0%

### Treatment planning under ultrasound guidance

Only 4.5 nodes (median value) were blocked due to the ultrasound probe (USP) position. Median PTV coverage without blocking the USP was 95.5% and 95.4% with USP blocking (note that patient 22 had a relatively unfavorable esophagus location). The V_9Gy _of the esophagus was increased by 1.9 cc with the USP block in one patient, but other visible changes in OAR dosimetry were not observed. Details are presented in Table 4.

**Table 4:** Ultrasound probe block optimization results PTV = Planning Target Volume, LCA = Left Coronary Artery, DMax = Maximum Dose, MU = Monitor Units, VxGy = Volume receiving X Gy or more

		PTV	Esophgaus		Bronchial Tree		LCA			
		Coverage	DMax	V9	DMax	V9	DMax			
Patient	Blocked	(%)	(Gy)	(cc)	(Gy)	(cc)	(Gy)	Nodes	Beams	MU
19	No	98.7%	18.1	8.2	18.7	5.1	12.9	89	264	28725
19	Yes	98.3%	17.8	8.5	19.1	5.3	12.9	86	262	27713
20	No	95.1%	18.2	7.8	21.1	23.8	12.5	92	257	29824
20	Yes	95.0%	18.1	9.7	21.3	22.8	12.5	87	242	28139
21	No	95.9%	19.0	2.5	19.1	5.8	12.3	93	245	29381
21	Yes	95.8%	19.0	3.0	19.5	6.5	12.5	88	228	27519
22	No	91.0%	19.6	6.7	19.4	2.7	12.8	92	255	29177
22	Yes	91.1%	19.9	6.4	19.6	2.2	13.0	88	249	28004
Median	No	95.5%	18.6	7.2	19.3	5.5	12.7	92.0	256.0	29279
Median	Yes	95.4%	18.6	7.5	19.6	5.9	12.7	87.5	245.5	27859

### Treatment planning under temporary fiducial guidance

**Figure 7:**
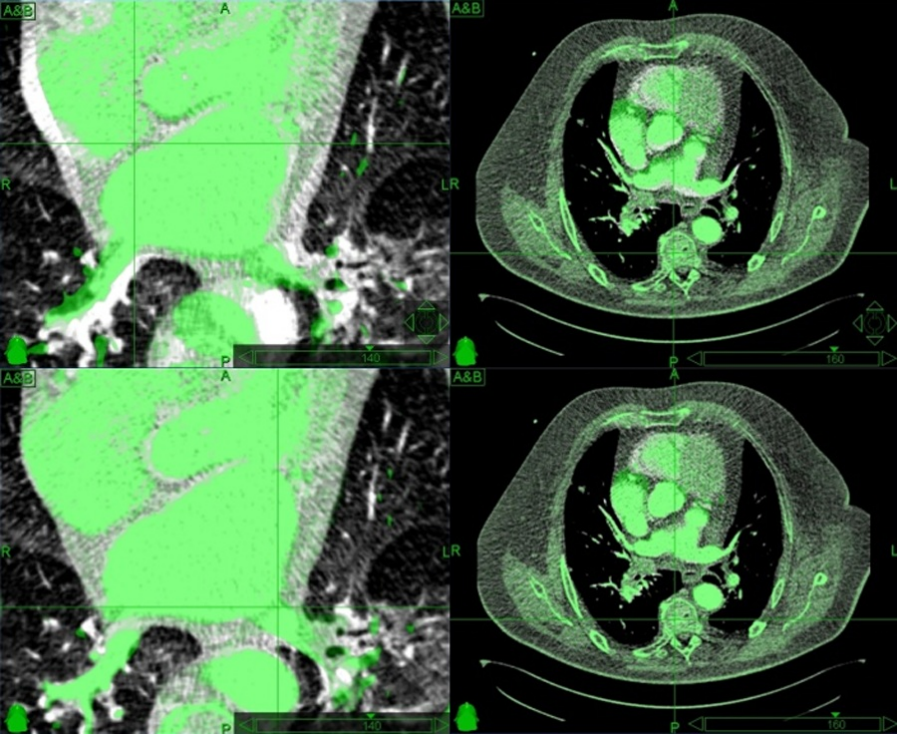
Alignment and deformation modeling of an example patient CT Differential motion over the respiratory cycle can be seen in the lower left and right pulmonary vein after alignment to the right atrial septum (top left). Maximum cardiac motion was approx. 1 cm in the right atrial septum (top right). Deformation modeling of respiratory (bottom left) and cardiac (bottom right) cycle was sufficient.

**Figure 8:**
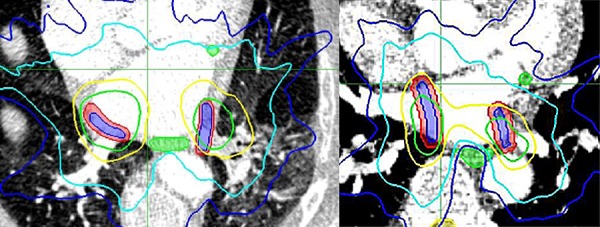
Examples of coverage reductions due to differential respiratory (left) and uncompensated cardiac (right) motion Blue = target lesions, red = planning target volumes, green = esophagus and coronary artery Isodose lines = 25 Gy (green), 12.5 Gy (yellow), 9 Gy (cyan), 6 Gy (blue)

Median respiratory and cardiac motion in the right atrial septum (theoretical fiducial location) was approximately 2 cm and 1 cm, respectively. The respiratory differential motion, especially between the right atrial septum and the left pulmonary vein, was clearly visible (Figure 7). Even though we had only two phases of the respiratory cycle (end expiration and end inspiration), the deformation model for both the respiratory and cardiac cycle was found to be sufficient using multiple landmarks (Table 5). Median coverage reduction for the 4D respiratory dose calculation of the static baseline plan was -5.7% for the RPTV and -17.6% for the LPTV. 4D dose optimization was able to reduce the reduction to less than -2.5% in all but one case (patient 24, LPTV coverage reduction -12%). Contrary to previous assumptions, we found substantial median coverage reductions of -23.4% for the RPTV and -13.2% for the LPTV in the 4D cardiac dose calculation. However, these results have to be taken with caution as the phase weighting of the 4D dose calculation in MultiPlan is optimized for respiratory motion which may not adequately reflect the actual cardiac motion. Examples of the coverage reductions are presented in Figure 8.

**Table 5:** 4D dose calculation and optimization results RPTV/LPTV = Right and Left Planning Target Volume, DMax = Maximum Dose RDC/CDC= Respiratory and Cardiac Dose Calculation, RDO = Respiratory Dose Optimization

Patient			23	24	25	26	Median
Atrial Septum Motion	Inferior/Superior	(mm)	10.4	15.2	8.0	21.6	12.8
	Anterior/Posterior	(mm)	15.0	13.6	3.0	13.0	13.3
	Left/Right	(mm)	5.1	3.0	6.4	2.4	4.1
	3D	(mm)	18.9	20.6	10.7	25.3	19.8
Respiratory Deformation	Landmarks		45	23	10	48	34
	Point Difference	(mm)	1.28	1.13	1.10	1.18	1.16
Cardiac Deformation	Landmarks		14	1	3	20	8.5
	Point Difference	(mm)	0.68	0.04	0.40	1.26	0.54
Baseline Plan	RPTV Coverage	(%)	99.5%	98.4%	95.4%	99.8%	99.0%
	LPTV Coverage	(%)	97.3%	95.4%	99.8%	98.5%	97.9%
	Esophgaus DMax	(Gy)	18.9	18.5	18.9	18.6	18.8
	Bronchial Tree DMax	(Gy)	20.4	20.9	21.5	19.3	20.7
4D RDC	RPTV Coverage Reduction	(%)	-7.1%	1.1%	-4.4%	-24.9%	-5.7%
	LPTV Coverage Reduction	(%)	-18.3%	-16.9%	-16.2%	-21.6%	-17.6%
	Esophgaus DMax Difference	(Gy)	-3.5	1.4	0.1	-3.4	-1.7
	Bronchial Tree DMax Difference	(Gy)	4.8	0.3	-2.0	-1.4	-0.6
4D RDO	RPTV Coverage Reduction	(%)	-1.3%	0.2%	0.1%	-0.3%	-0.1%
	LPTV Coverage Reduction	(%)	-2.1%	-12.0%	0.0%	-1.8%	-2.0%
	Esophgaus DMax Difference	(Gy)	0.0	0.6	-0.3	0.8	0.3
	Bronchial Tree DMax Difference	(Gy)	0.4	0.3	-0.1	0.9	0.4
4D CDC	RPTV Coverage Reduction	(%)	-36.0%	-1.5%	-10.7%	-46.1%	-23.4%
	LPTV Coverage Reduction	(%)	-18.5%	0.8%	-8.0%	-40.8%	-13.2%
	Esophgaus DMax Difference	(Gy)	-0.3	1.3	-0.3	-0.5	-0.3
	Bronchial Tree DMax Difference	(Gy)	-1.4	1.6	0.3	-4.0	-0.5

## Discussion

We analyzed treatment planning with a prescription dose of 25 Gy for atrial fibrillation (AF) robotic radiosurgery given several potential tracking options. We found that with currently known guidelines on critical structure dose limitations [41, 42, 52, 53], treatment planning under ideal conditions, i.e. treatment accuracy below 3 mm, may only be feasible in less than 40% of the patients. This is mainly due to an unfavorable esophagus and bronchial tree location relative to the left atrial antrum [30-34] where the lesions would have to be placed to treat AF [12, 13, 24-29]. This feasibility analysis was conducted under the assumption that 25 Gy prescription dose would be sufficient to block conduction from the pulmonary veins (PV) to the left atrium (LA). This is of great debate as some studies suggested that 25 Gy may be sufficient [5, 6, 9] whereas others found that with 25 Gy only little effects are seen [10] and that more than 30 Gy are needed [7, 8, 54]. This may further reduce the number of suitable patients for AF radiosurgery.

Most of the dose-response modeling was derived from animal studies [5-8], and only limited patient experiences are available today [9, 10]. One problem for translating dose modeling from animals into humans is that in humans all PVs would have to be electrically blocked to treat AF whereas in previous animal studies only a limited number of PVs were treated. This translated into relatively short treatment times in the animal studies, even with the robotic CyberKnife. For larger non-cancerous target areas, especially when treated with the small CyberKnife beams, cell-repair rates during treatment may have to be considered [45, 46]. For the first time, we analyzed the regional dose rate (RDR) of a CyberKnife treatment delivery and found substantial optimization possibilities when sequentially delivering beams to specific parts of the target lesions versus delivering all beams in one pre-defined path; as usually done with the Iris collimator [14, 39]. The same problem may appear during routine tumor treatment, e.g. when treating multiple or large complex target lesions with treatment times over one hour, even though studies suggested that tumor cells are not as prone to cell repair as healthy cells during pulsed irradiation [43, 44]. While this warrants further investigation, the problem of long treatment times with the CyberKnife may be solved altogether using a multi-leaf collimator (MLC) for robotic radiosurgery [55], but planning with the MLC was not available at the time this study was performed.

Regardless of the prescription dose or the RDR, it is obvious that the treatment delivery accuracy for AF radiosurgery has to be kept as high as possible. Treatment planning studies for MRI-guided linear accelerator based AF radiosurgery found that 3 mm is the maximum tolerable margin for a 30 Gy treatment [18] and based on our initial experience we would agree this to be the same for robotic guided AF radiosurgery (3-5 mm safety margin for a 25 Gy to 30 Gy AF radiosurgery treatment). Unfortunately, one drawback of the CyberKnife in its present state is the necessity to rely on x-ray imaging and external motion correlation. Direct marker-less PV tracking on the 45 degree orientated x-rays was found to be infeasible, hence “temporary” fiducial implantation seems to be a valid alternative solution [10, 11]. However, in contrast to ventricular tachycardia previously treated with cardiac SRS [10, 11], the AF target area is relatively large, and a differential surrogate-to-target motion may arise. This behavior was validated in our small treatment planning study on unique 4D cardiac CT in end expiration and end inspiration. The differential motion may be compensated for by using 4D Planning [51] or even 5D planning [54], combining respiratory and cardiac motion alike, but the repeatability of 4D or 5D planning during treatment may be questionable and further studies are warranted.

Concluding from this initial planning study in a relatively small cohort, we believe that direct real-time target tracking would be necessary for AF radiosurgery not to invalidate the non-invasive idea of cardiac radiosurgery. Currently, MRI guidance [18] and ultrasound [21, 22, 47, 48] offer direct target visibility without using ionizing radiation and are therefore the most promising options. Especially ultrasound has high potential to be integrated into the CyberKnife [22, 47] as we found negligible treatment plan quality reductions when blocking the ultrasound probe placed on the patients’ chest, warranting further confirming studies.

Limitations with all their associated problems to this study are the number of analyzed data sets, especially for an ultrasound and differential motion planning, the mixed presentation of the data, the extreme patient selection with favorable esophagus location, and the analysis with only one prescription dose. Random effects in treatment planning and subsequent plan comparison may be small [38-40], but cannot be disregarded in this sample size. 4D planning comes with all its known problems [49-51] and 5D dose calculation [54] or phantom studies [36] may be more appropriate when investigating dosimetric effects from cardiac motion. Advanced tuning for CyberKnife treatment planning [56, 57] and using the MLC [55] have not been considered as those were unavailable at the time this study was performed, but may potentially increase the possible number of treatable patients.

One remaining concern to be raised for AF radiosurgery is the relatively large 10 Gy volume of the whole heart. While doses above 35 Gy may induce significant side effects such as myocarditis, cardiovascular disease and pneumonitis [58, 59], long-term toxicity of lower doses may need further investigation. Side effects to the heart may occur years after treatment [60] with a potential increase in the risk of radiation-induced coronary events (RICE) [61]. These long-term risks could be overestimated for single fraction treatments [62], and are for arrhythmia patients at high age, potentially irrelevant. Great care should still be taken during patient selection for potential AF radiosurgery, and dose-volume effects in the heart should be considered [63].

## Conclusions

Robotic AF radiosurgery with 25 Gy may be feasible in a subgroup of patients under ideal tracking conditions. Ultrasound tracking was found to have the least impacts on treatment planning and given its real-time imaging capability should be considered for AF robotic radiosurgery. Nevertheless, advanced treatment planning using regional dose rate or 4D respiratory and cardiac dose optimization may still be advised despite using ideal tracking methods.
